# Silencing NADPH-cytochrome P450 reductase results in reduced acaricide resistance in *Tetranychus cinnabarinus* (Boisduval)

**DOI:** 10.1038/srep15581

**Published:** 2015-10-23

**Authors:** Li Shi, Jiao Zhang, Guangmao Shen, Zhifeng Xu, Peng Wei, Yichao Zhang, Qiang Xu, Lin He

**Affiliations:** 1 Key Laboratory of Entomology and Pest Control Engineering, College of Plant Protection, Southwest University, Chongqing, China; 2 Department of Biology, Abilene Christian University, Abilene, Texas, USA

## Abstract

Cytochrome P450 monooxygenases (P450s) are involved in metabolic resistance to insecticides and require NADPH cytochrome P450 reductase (CPR) to transfer electrons when they catalyze oxidation reactions. The carmine spider mite, *Tetranychus cinnabarinus* is an important pest mite of crop and vegetable plants worldwide, and its resistance to acaricides has quickly developed. However, the role of CPR on the formation of acaricide-resistance in *T. cinnabarinus* is still unclear. In this study, a full-length cDNA encoding CPR was cloned and characterized from *T. cinnabarinus* (designated *TcCPR*). *TcCPR* expression was detectable in all developmental stages of *T. cinnabarinus*, but it’s much lower in eggs. *TcCPR* was up-regulated and more inducible with fenpropathrin treatment in the fenpropathrin-resistant (FeR) strain compared with the susceptible SS strain. Feeding of double-strand RNA was effective in silencing the transcription of *TcCPR* in *T. cinnabarinus*, which resulted in decreasing the activity of P450s and increasing the susceptibility to fenpropathrin in the FeR strain but not in the susceptible strain. The current results provide first evidence that the down-regulation of *TcCPR* contributed to an increase of the susceptibility to fenpropathrin in resistant mites. TcCPR could be considered as a novel target for the development of new pesticides.

The carmine spider mite, *Tetranychus cinnabarinus*, is one of the major pest species of agriculture crops in China^1^. It enjoys worldwide distribution with more than 100 crops or plants grown in the field or greenhouse^2^. Since its morphological, biological, and molecular characteristics are quite similar to those of the two-spotted mite, *Tetranychus urticae*, many researchers also considered them as two forms (red and green) of a single species (*T. urticae*)^3^,^4^,^5^. In recent years, the intensive use of insecticides and acaricides has led to resistance in many insect and mite species around the globe. The effort to control resistant insects and mites is becoming exceedingly challenging^5^,^6^.

Cytochrome P450 monooxygenases (P450s) belong to a superfamily of hemecontaining enzymes that catalyze the monooxygenation of xenobiotics and endogenous compounds. They play important roles in metabolic resistance of agricultural insects and mites^7^. Many studies suggested that the over-expression of P450s was involved in metabolism of pesticides. Functional study of *CYP392E10*^8^ and *CYP392A16*^9^ in resistant strains of *T. urticae* confirmed that they could metabolize spirodiclofen and abamectin, respectively. Yang *et al.*^10^ also reported that *CYP9A12* and *CYP9A14* were responsible for pyrethroid resistance in *H. armigera.* NADPH-cytochrome P450 reductase (CPR) is an essential enzyme in all kingdoms of life and is involved in a variety of biological reactions catalyzed by microsomal P450s^11^,^12^. Although multiple P450 genes have been found in insect genomes, so far, only one CPR gene has been identified in each insect or mite genome. CPR is a diflavin enzyme that contains domains for Flavin Mononucleotide (FMN), Flavin Adenine Dinucleotide (FAD), and Nicotinamide Adenine Dinucleotide Phosphate (NADP) binding^13^ (Fig. 1A). The reaction of the P450s system requires electrons transferred from Nicotinamide Adenine Dinucleotide Phosphate (NADPH) to the P450 heme center by CPR^14^. In addition to cytochrome P450s, CPR also serves as the electron donor protein for several oxygenase enzymes found in the endoplasmic reticulum of most eukaryotic cells^15^,^16^.

As inhibition of CPR effectively shuts down all microsomal P450 activities, this provides a route for the establishment of P450s’ contribution to metabolic detoxification in complex organisms^17^. RNA interference (RNAi) is a universal gene-silencing mechanism in eukaryotic organisms. RNAi-based technology has shown great potential in controlling insect pests by silencing vital genes^18^,^19^. Recent studies have demonstrated that silencing CPR by RNAi in *Cimex lectularius*^20^ and *Anopheles gambiae*^21^ increases susceptibility to pyrethroid insecticides, revealing an essential role for CPR in P450s-mediated biochemical processes in insects, and it would be of great interest to develop synthetic inhibitors targeting this gene for the use in insect pest management.

In the current study, a full-length cDNA of the CPR from *T. cinnabarinus* (*TcCPR*) was cloned and characterized, and the gene expression patterns in different developmental stages, in different strains, and upon acaricide induction were analyzed. Here, we have also adopted dsRNA feeding as a gene-knockdown strategy to investigate the role of CPR in detoxification function of P450s in *T. cinnabarinus*. This study indicates that the suppression of *TcCPR* transcription contributes to an increase of acaricide susceptibility in resistant mites and helps to reveal the role of P450-mediated metabolic detoxification in the fenpropathrin-resistance in *T. cinnabarinus* (Fig. 1B).

## Results

### cDNA cloning and characterization of *TcCPR*

**Figure 1:**
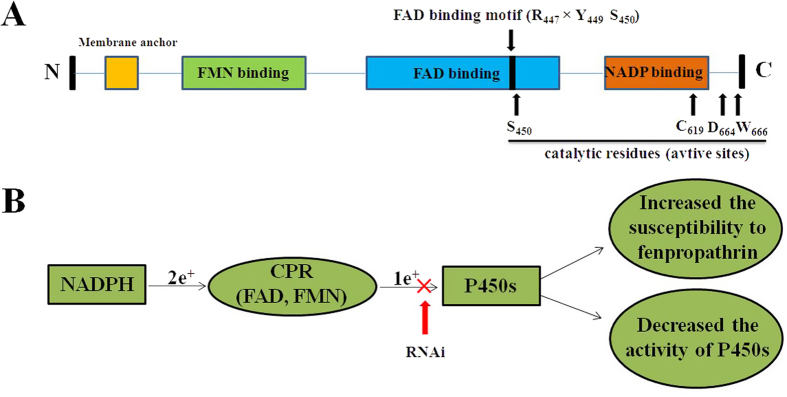
(**A**) Schematic drawing of *TcCPR*; (**B**) The role of *TcCPR* on P450 activities and acaricide resistance in *Tetranychus cinnabarinus*.

Using our transcriptome data, we were able to identify and clone the coding sequence containing the complete ORF of *TcCPR* in this study. A full-length cDNA sequence of *TcCPR* was isolated (GenBank accession number: KP710970). *TcCPR* contained an ORF of 2004 bp and encoded a deduced peptide of 667 amino acid residues with predicted MW and pI of 75.47 kDa and 5.70, respectively. A signal peptide with 26 amino acid residues was found at the N-terminal end of *TcCPR*, and a membrane anchor with 23 amino acid residues that facilitates the localization of *TcCPR* on the endoplasmic reticulum was identified. All functional domains involved in the binding of cofactors, such as FMN and FAD, and involved in the receiving electrons from NADPH were identified in the predicted *TcCPR* protein primary and tertiary structures. These regions have been reported to the binding sites of the coenzymes^22^. The FAD binding motif of *TcCPR* consisted of three amino acid residues (Arg 447, Tyr 449, and Ser 450) (Fig. 1A). Meanwhile, two FMN binding sites were identified at the N-terminal of the FMN domain (Fig. 1A). The catalytic residues of *TcCPR* were comprised of Ser 450, Cys 619, Asp 664, and Try 666 (Fig. 1A). These residues have been documented to be critical in the hydride transfer reaction catalyzed by rat CYP oxidoreductase^23^,^24^.

### Phylogenetic analysis of *TcCPR*

**Figure 2:**
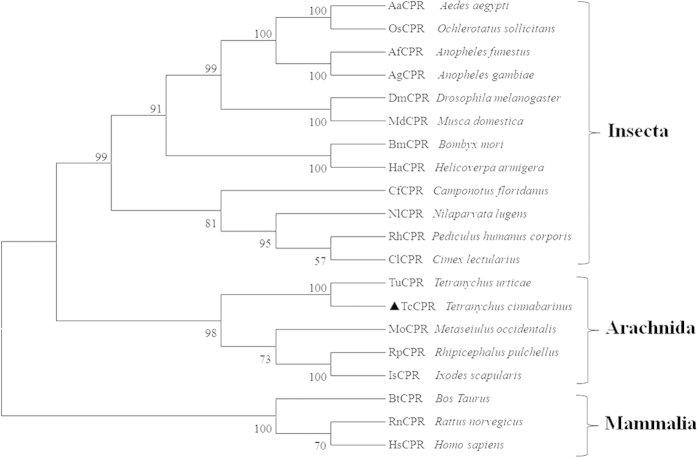
Phylogenic analysis of CPRs. The maximum likelihood tree was constructed by MEGA 5.05. The testing of phylogeny was done by the bootstrap method with 1000 replications. The sequences used for constructing the tree are listed in Supplementary Table S2.

Phylogenetic analysis was performed by MEGA 5.05 with the maximum likelihood method on the basis of the deduced amino acid sequences of *TcCPR* and other known CPR proteins, including orthologs from arachnids, insects, and mammals. All CPR sequences, which have the complete ORFs, were obtained from the National Center for Biotechnology Information (NCBI) (Bethesda, MD) (http://www.ncbi.nlm.nih.gov/) (Supplementary Table S2). The result showed that *TcCPR* was grouped to arachnida and shared the highest sequence similarity with the CPR of *T. urticae* (*TuCPR*) (Fig. 2), suggesting evolutionary relatedness and possibly similar physiological functions between *TcCPR* and *TuCPR*.

### Expression patterns of *TcCPR* in different developmental stages, in different strains, and upon acaricide induction

**Figure 3:**
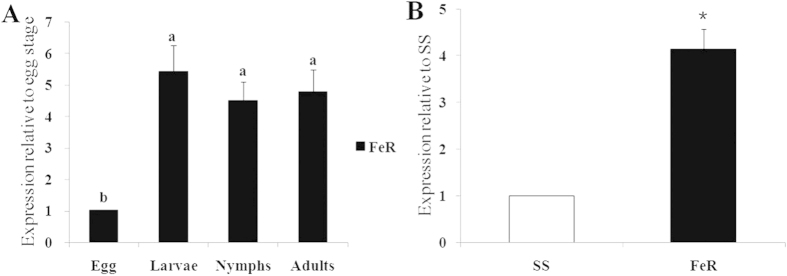
Quantitative PCR expression analysis of *TcCPR*. (**A**) qPCR analysis of *TcCPR* expression in different life stages of FeR strain, relative to the expression level in eggs. (**B**) qPCR analysis of *TcCPR* expression in different strains. Error bars represent the standard error of the calculated mean based on three biological replicates. The asterisk indicates significant difference according to independent-sample *t*-test (*P* < 0.05). Different letters on the error bars show significant difference according to Duncan’s multiple tests (*P* < 0.05). i.e. No statistical difference between “a” and “a”; significant difference between “a” and “b”.

**Figure 4:**
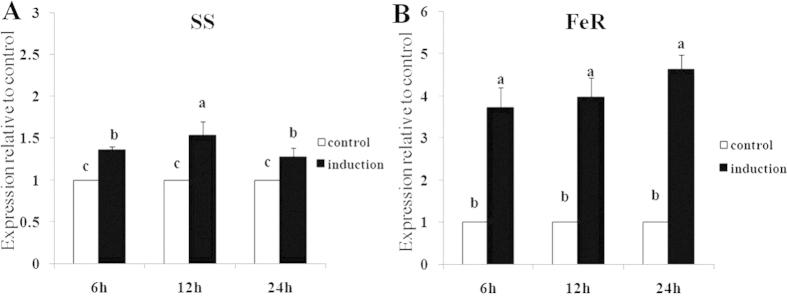
Expression profiles of the induction of *TcCPR* in SS and FeR strains after fenpropathrin treatment. (**A**) Expression levels of *TcCPR* in the SS strain. (**B**) Expression levels of *TcCPR* in the FeR strain. Error bars represent the standard error of the calculated mean based on three biological replicates. Different letters on the error bars show significant difference according to Duncan’s multiple tests (*P* < 0.05). i.e. No statistical difference between “a” and “a”; significant difference between “a” and “b”, between “b” and “c”, and between “a” and “c”.

The expressions of the *TcCPR* gene in different developmental stages, in different strains, and upon acaricide induction were investigated using quantitative reverse-transcriptase PCR (qRT-PCR). The results showed that expression levels of *TcCPR* were not significantly different among different development stages, except for eggs, which had a much lower expression level. The expression levels of *TcCPR* in larvae, nymphs, and adults were 5.40-, 4.47-, and 4.75-fold of the expression level in eggs (Fig. 3A). Identification of the expression difference of *TcCPR* between different strains indicated that it was over-expressed in FeR strain, in which it was significantly up-regulated to 4.12-fold compared with susceptible SS strain (Fig. 3B). The results of fenpropathrin induction experiment revealed that the *TcCPR* gene was up-regulated both in SS and FeR after treated with fenpropathrin. The relative expression levels of *TcCPR* in FeR strain increased to 3.70-, 3.95-, and 4.61-fold compared with control after 6-, 12-, and 24-hours of fenpropathrin induction, respectively (Fig. 4B). However, a much lower level (<1.50-fold) of induction for *TcCPR* was detected in the susceptible SS mites during different time frames (Fig. 4A).

### Silencing of *TcCPR* by RNAi

**Figure 5:**
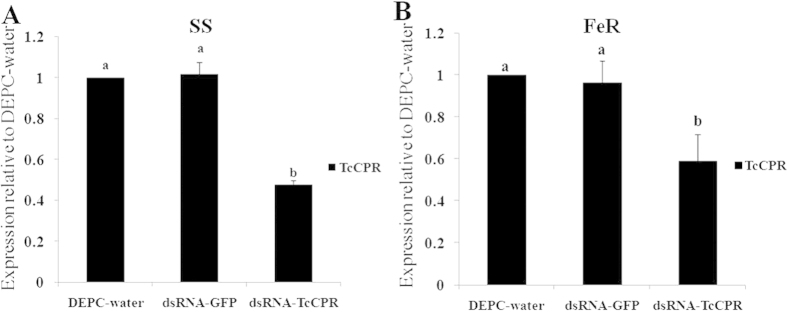
Quantitative PCR expression analysis of *TcCPR* after dsRNA-*TcCPR* feeding, relative to the expression levels after DEPC-water treatment. (**A**) Expression levels of *TcCPR* in SS strain. (**B**) Expression levels of *TcCPR* in FeR strain. Error bars represent the standard error of the calculated mean based on three biological replicates. Different letters on the error bars show significant difference according to Duncan’s multiple tests (*P* < 0.05). i.e. No statistical difference between “a” and “a”; significant difference between “a” and “b”.

**Figure 6:**
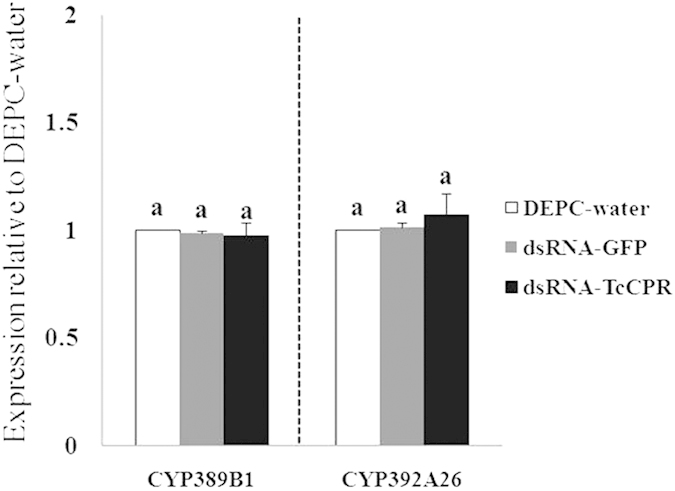
Quantitative PCR expression analysis of two P450 genes after dsRNA-*TcCPR* feeding. Error bars represent the standard error of the calculated mean based on three biological replicates. Different letters on the error bars show significant difference according to Duncan’s multiple tests (*P* < 0.05). i.e. No statistical difference between “a” and “a”.

To verify the effectiveness of RNAi, approximately 200 female adults from different strains were collected after feeding for 48 hours, and the experiments were replicated three times. The qRT-PCR data showed that the *TcCPR* expression levels decreased significantly to 47.5% in the SS strain and to 59% in the FeR strain 48 h after feeding of dsRNA-*TcCPR* compared with feeding of DEPC-water or dsRNA-*GFP* (Fig. 5), indicating that feeding of dsRNA of *TcCPR* was effective in silencing the transcription of *TcCPR*. Interestingly, DEPC-water, dsRNA-*GFP* and dsRNA-*TcCPR* treatments had no significantly impact on the expression of *CYP389B1* as well as *CYP392A26* (Fig. 6), suggesting that down-regulating *TcCPR* with dsRNA-*TcCPR* feeding would not impact P450 gene expressions.

### Assay of P450 activities after RNAi

Results of specific activity changes of P450s after RNAi of the *TcCPR* gene in *T. cinnabarinus* were shown in Table 1. The P450s specific activity decreased significantly 48 h after feeding of dsRNA-*TcCPR* compared with DEPC-water or dsRNA-*GFP* feeding. These results suggested that *TcCPR* played a central role in P450 activities in *T. cinnabarinus* and that *TcCPR* was a key factor of P450s in conferring metabolic resistance against acaricide in mites.

### Detection the sensitivity to fenpropathrin after *TcCPR* knockdown

**Figure 7:**
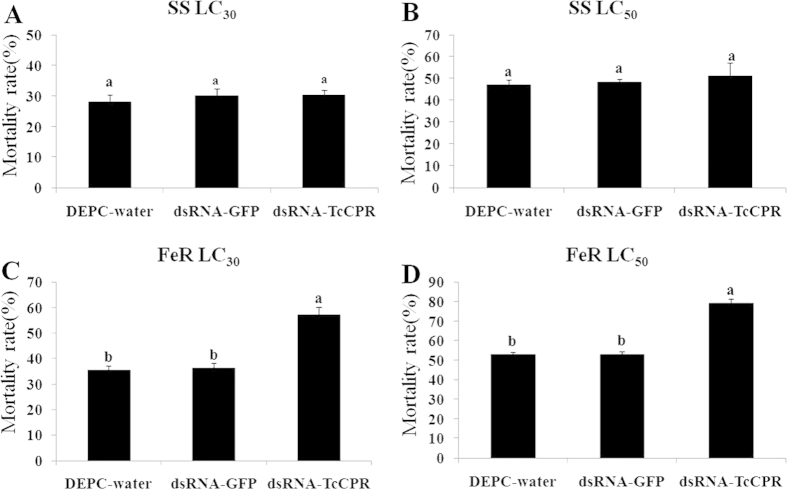
Knockdown of *TcCPR* expression reduced the resistance to fenpropathrin in mites. (**A**,**B**) Mortality of *TcCPR*-silenced *T. cinnabarinus* to fenpropathrin at LC_30_ and LC_50_ in the SS strain, respectively. (**C**,**D**) Mortality of *TcCPR*-silenced *T. cinnabarinus* to fenpropathrin at LC_30_ and LC_50_ in the FeR strain, respectively. Error bars represent the standard error of the calculated mean based on three biological replicates. Different letters on the error bars show significant difference according to Duncan’s multiple tests (*P* < 0.05). i.e. No statistical difference between “a” and “a”; significant difference between “a” and “b”.

Knockdown of *TcCPR* via RNAi in the female adults of *T. cinnabarinus* significantly increased their susceptibility to fenpropathrin compared with DEPC-water and dsRNA-*GFP* treatments. The mortality increased significantly from 35.53% and 53.09% in the control to 57.11% and 79.28% in the dsRNA-*TcCPR*-fed fenpropathrin resistant FeR strain when treated with LC_30_ and LC_50_, respectively (Fig. 7C,D). The mortality showed no significant difference between DEPC-water and dsRNA-*GFP* treatments (Fig. 7C,D). In contrast, there was no significant difference in the susceptibility to fenpropathrin between *TcCPR* knockdown treatment and controls in susceptible SS strain (Fig. 7A,B). These results revealed that the down-regulation of *TcCPR* has a crucial impact on the toxicity of fenpropathrin to resistant *T. cinnabarinus*, which increased the mites’ sensitivity to fenpropathrin.

## Discussion

Insecticide resistance in insects and mites has been attributed to increased rates of insecticide detoxification or mutations at target sites^25^,^26^. The mechanisms mediating resistance to pyrethroids have been studied intensively in insects and mites. Some mutations in the sodium channel gene have been shown to confer target site insensitivity to the neurotoxic effects of pyrethroids^27^. Tsagkarakou *et al.*^28^ identified the point mutation (F1538I) in *T. urticae*, and Feng *et al.*^29^ also has reported the same mutation (F1538I) in the sodium channel gene of *T. cinnabarinus* fenpropathrin-resistant strain, which is known to confer high resistance to pyrethroids. Among the elucidated metabolic mechanisms of resistance, the most common ones include enhanced detoxification of pyrethroids by overexpressed P450s, CarEs and GSTs^30^,^31^,^32^. In our previous study, we demonstrated that the increase of the activities of P450s was important in conferring fenpropathrin resistance in *T. cinnabarinus*^33^.

P450s play crucial roles in the development of insecticide resistance in insects and mites by degrading pesticides before they reach the target sites. P450s are components and key factors of mixed function oxidases (MFOs). The exertion for their function requires the cytochrome P450 reductase (CPR) to transfer electrons from NADPH to the substrate complex so that they can catalyze the metabolism of a variety of substrates including endogenous compounds and xenobiotics^34^,^35^. Consequently, identification and characterization of CPR from insects and mites will be helpful to determine whether or not P450s are involved in responses of insects to specific insecticides and other xenobiotics^21^,^36^, and might help us identify new targets for the development of chemical synergists. However, compared with P450s, the documented research on the role of CPR involved in the formation of insecticide-resistance is far from enough.

In this study, we cloned a full-length cDNA of CPR gene in *T. cinnabarinus* (designated *TcCPR*). The structure analysis of *TcCPR* demonstrated that this gene has a signal peptide region and four functional domains. The hydrophobic N-terminal membrane anchor of *TcCPR* is essential for its function in the P450 catalytic cycle. It serves to anchor the protein molecule to the endoplasmic reticulum that ensures proper spatial interaction for electron transfer between the *TcCPR* and P450s^35^,^37^. Therefore, without the hydrophobic anchor, *TcCPR* may be incapable of transferring electrons to P450s and other receptors in *T. cinnabarinus*. Three conserved binding domains, a FAD binding motif, and the catalytic residues as well as the critical residues involved in FMN, FAD, and NADP binding were identified. These binding sites were well conserved among all insect CPRs, indicating that they play key roles in the interaction with P450s. Lamb *et al.*^38^ demonstrated that mutations of amino acid residues Asp 187 and Thr 71 to Ala in the yeast CPR resulted in complete loss of functional activity toward *CYP51*.

P450s of insects and mites catalyze a diverse array of metabolic reactions during their whole life cycle. It has been reported that dramatic variations occur in the expression levels of P450s during the development of most insects, either within or between life stages^39^. Catalytic activities of P450s require involvement of their redox partner, CPR. Therefore, development-related expression of the *TcCPR* gene is a reflection of P450 activity in *T. cinnabarinus*. Our present results showed that expression level of *TcCPR* was not significantly different among different developmental stages but was much lower during the egg stage. This is remarkable as it is similar to the expression patterns of two P450 genes, *CYP389B1* and *CYP392A26*, in the FeR strain of *T. cinnabarinus*^40^. This phenomenon was also observed for *CYP392E7* and *CYP392E10* in the spirodiclofen-resistant strain of *T. urticae*^8^, in which the expression levels were high during late developmental stages but almost absent during the egg stage^41^. The wide distribution and expression of *TcCPR* across different developmental stages of *T. cinnabarinus* probably indicate its involvement in various demands of different P450s for driving biosynthesis and metabolic processes, which are not in high demand during the egg stage. Meanwhile, the level of *TcCPR* expression in the FeR strain was significantly higher than that in the SS strain, which supports our previous findings that P450 genes were over-expressed in the FeR strain of *T. cinnabarinus*^40^. The up-regulation of CPR expression might result in an increase of its capacity of electron transfer, which is necessary for the enhancement of the oxidizing ability of P450s. Co-expression of P450 gene *CYP392A16* and CPR of *T. urticae* in *E.coli* confirmed that the recombinant protein could metabolize abamectin, which also indicates that CPR plays a central role for numerous biological reactions catalyzed by P450s^9^.

Several studies have shown that CPRs were inducible by drugs in other organisms. For instance, the level of CPR mRNA in *Helicoverpa armigera* was up-regulated in the midgut of 6th instar larvae after treated with phenobarbital (PB) in the diet^42^. Shephard *et al.*^43^ reported that the activity of the CPR enzyme in rat liver microsomal membranes increased to 2.7- fold after PB treatment. Our present study showed that, after exposure to fenpropathrin, the expression levels of *TcCPR* were increased both in SS and FeR strains, and that the induction of *TcCPR* expression to fenpropathrin challenge in resistant FeR strain was much more significant than the induction in susceptible SS strain. These results were also similar to the fenpropathrin-induced expression patterns of two P450 genes, *CYP389B1* and *CYP392A26*, in fenpropathrin-resistant and susceptible *T. cinnabarinus*^40^, which suggested that *TcCPR* plays an essential role in P450-mediated resistance against fenpropathrin in *T. cinnabarinus*.

In order to effectively carry out the RNAi experiment, we adopted a method of leaf-disc feeding containing dsRNA-*TcCPR* to knock down the expression of *TcCPR* in *T. cinnabarinus*. The results of qRT-PCR analyses showed that *TcCPR* transcript levels significantly decreased to 40–50% in mites 48 h after fed with dsRNA-*TcCPR*. The P450 activities also decreased more than 4 folds in the FeR strain after feeding of dsRNA-*TcCPR*. These results not only indicated that the dsRNA-mediated knockdown of *TcCPR* transcript was successful but also revealed that *TcCPR* is a key factor for function exertion of P450s in *T. cinnabarinus*. Furthermore, the results also demonstrate that the delivery of dsRNA via leaf-disc can now be considered as a suitable method to achieve RNAi in *T. cinnabarinus*. However, the expression levels of two P450 genes (*CYP389B1* and *CYP392A26*) were not changed at 48 h post-feeding of dsRNA-*TcCPR*, which suggested that CPR is not a regulator of P450 expression. A decrease of P450 activity observed in this study was not because of the down-regulation of P450 expression but because of the RNAi of *TcCPR* resulting in a decline of the efficiency of transferring electrons to P450s in *T. cinnabarinus*.

In insects and mites, overexpression of P450 genes is often involved in enhanced detoxification of insecticides or plant secondary metabolites^26^. Inactivation of P450 or its redox partner, CPR, by the application of inhibitors or knockdown target genes using RNAi, results in an increase of susceptibility to synthetic and natural compounds. Inactivation of CPR as a whole has been documented to increase the deltamethrin susceptibility in *Cimex lectularius*^20^ and the permethrin susceptibility in *Anopheles gambiae*^21^. Our results showed that *TcCPR* contributed in a similar manner. That is, the susceptibility to fenpropathrin increased when the *TcCPR* was suppressed through RNAi in fenpropathrin-resistant *T. cinnabarinus*. More interestingly, the susceptibility to fenpropathrin was not impacted in susceptible mites when the *TcCPR* expression level was knocked down for more than 50%, suggesting that, in susceptible SS strain, most P450 gene expressions were not involved in metabolic detoxification of acaricides. These results suggest that the P450/CPR complex could be an important factor involved in the detoxification of fenpropathrin and that CPR is also crucial to the functional exertion of P450s, such as mediating metabolic resistance in *T. cinnabarinus*. This study is the first report in *T. cinnabarinus* that knocking down CPR gene expression decreased the activity of P450s and the resistance to fenpropathrin in resistant mites.

In conclusion, the present study provides preliminary information on the sequence, phylogenicity, and expression pattern of *TcCPR* gene in *T. cinnabarinus. TcCPR* expressed in a similar pattern in late developmental stages with a higher level than in eggs. Specific expression detection showed that *TcCPR* was overexpressed and was more inducible with the treatment of fenpropathrin in FeR strain compared with the susceptible strain. More importantly, the down-regulation of *TcCPR* decreased the activity of P450s and increased susceptibility to fenpropathrin in resistant FeR strain but not in susceptible SS strain. This study provides valuable information on the correlation between P450/CPR complex and acaricide-resistance in mites. TcCPR might be considered as a novel target for the development of new pesticides.

## Methods

### Mites

Susceptible strain (SS): the laboratory carmine spider mite population was originally collected from the field in Beibei District, Chongqing, China. Then, it was transferred to fresh potted cowpea leaves and kept in artificial climate chamber (pesticide free) for more than 15 years. FeR strain: the resistant strain was selected with fenpropathrin for >70 generations in the laboratory from the SS strain. The detailed information on the selection procedure refers to He *et al.*^33^. *Tetranychus cinnabarinus* mites were reared at 26 ± 1 °C temperature, 35%–55% relative humidity (RH), and a photoperiod of 14:10 h (L:D).

### Reagents

The 92% fenpropathrin was purchased from Bangnong Chemical Company (Guangzhou, China); p-nitrophenol and p-nitroanisole were obtained from Shanghai SSS Reagent Co. (Shanghai, China); coenzyme NADPH and hydroxymethyl aminomethane (Tris) were from Shanghai Dingguo Biotech Development Co. (Shanghai, China); Coomassie blue G-250 was acquired from Amresco Co. (Solon, USA); bovine serum albumin (BSA) was from Shanghai BioLife Science & Technology Co. (Shanghai, China); other chemicals and reagents were the highest quality commercially available from local suppliers.

### Total RNA isolation, synthesis of cDNA and cloning of *TcCPR*

Total RNAs were isolated from 200 female adults (3–5d old) of *T. cinnabarinus* using RNeasy^®^ plus Micro Kit (Tiangen, Beijing, China). To check the RNA quantity, the absorbance at 260 nm and the absorbance ratio of OD_260/280_ were measured with a Nanovue UV-Vis spectrophotometer (GE Healthcare, Fairfield, CT). The reverse transcription was carried out using PrimeScript^®^ 1st Strand cDNA Synthesis Kit (Takara Biotechnology Dalian Co., Ltd., Dalian, China) and the synthesized cDNAs were stored at −20 °C.

In the transcriptome data of *T. cinnabarinus*, we have identified the full encoding sequence of *TcCPR*^44^. Thus, we performed PCR to verify the opening reading frame (ORF) of *TcCPR*. The primers were presented in Supplementary Table S1. Specific PCR reaction was performed in a C1000™ Thermal Cycler (BIO-RAD, Hercules, CA, USA). The amplified PCR fragments were gel-purified with the Gel Extraction Mini Kit (Watson Biotechnologies, Shanghai, China), ligated into pGEM-Teasy vector (Promega, Fitchburg, MA, USA), and then transformed into *Escherichia coli* Transα competent cells (TransGen Biotech, Beijing, China). Cloning and sequence analyses of *TcCPR* gene fragments were repeated at least three times with different preparations of RNAs. Inserts were further sequenced for confirmation (BGI, Beijing, China).

### Bioinformatic analyses

DNA sequences were edited with DNAMAN 5.2.2. The ClustalW program was used to align the deduced amino acid sequence of *TcCPR*^45^. The isoelectric point (pI) and molecular weight (MW) of *TcCPR* were calculated by an ExPASy proteomics tool (http://cn.expasy.org/tools/pi_tool.html)^46^. The signal peptide was predicted using Signa1P 3.0 (http://www.cbs.dtu.dk/service/SignalP/)^47^. The transmembrane helices of *TcCPR* were analyzed by TMHMM Server 2.0 (http://www.cbs.dtu.dk/services/TMHMM-2.0/).

The binding domains and catalytic residues were predicted by Conserved Domain Search (www.ncbi.nlm.nih.gov/Structure/cdd/cdd.shtml)^48^. A phylogenetic tree was constructed by the maximum likelihood method using MEGA 5.05 software^49^. A total of 1000 bootstrap replications were used to test the phylogeny.

### qRT-PCR of *TcCPR*

The primers used for qRT-PCR of *TcCPR* were designed by using primer 3.0 (http://frodo.wi.mit.edu/)^50^. *RPS18* (FJ608659) and *α-TUB*(FJ526336) were used as stable reference genes to all qRT-PCR assays (Supplementary Table S1)^51^. In order to detect the expression of *TcCPR* in different life stages in the FeR strain, approximately 2000 eggs, 1000 larvae, 800 nymphs, and 200 adults from the FeR strain were collected for each sample. We collected 200 female adults in SS and FeR strains for each sample to quantify the expression of *TcCPR* in different strains. To examine the effect of fenpropathrin exposure on the expression of *TcCPR*, fenpropathrin (LC_30_) was used to treat SS and FeR strain female adults for 6, 12 and 24 h, respectively. For the induction experiment, we adopted the modified residual coated vial (RCV) method recommended by Van Leeuwen *et al.*^52^,^53^. After the interval, only surviving female adults from treated and control groups were collected for RNA extractions. Each experiment was repeated at least three times using independent biological samples. The qRT-PCR was performed on an Mx3000P thermal cycler (Agilent Technologies, Inc., Wilmington, NC, USA) with 20 μL reaction mixtures containing 1 μL cDNA, 10 μL iQ™ SYBR® Green Supermix (BIO-RAD, Hercules, CA, USA), 1 μL of each gene-specific primer (0.2 mM), and 7 μL ddH_2_O. The optimized qRT-PCR protocol used for the amplification was: 95 °C for 2 min, then 40 cycles of denaturation at 95 °C for 15 s, annealing at 60 °C for 30 s, and elongation at 72 °C for 30 s. Finally, melt curve analyses (from 60 to 95 °C) were included at the end to ensure the consistency of the amplified products. The expression level was calculated using the 2^−ΔΔCt^ method^54^ and normalized by the average of two reference genes.

### dsRNA feeding and silencing of *TcCPR* by RNAi

*TcCPR* and the Green Fluorescent Protein (*GFP*) gene were amplified by PCR using primers containing T7 RNA polymerase promoter (Supplementary Table S1). Products were gel purified and used as templates to synthesize dsRNAs using TranscriptAid T7 High Yield Transcription Kit (Thermo scientific, Lithuania, EU). The dsRNAs were further purified using the GeneJET RNA Purification Kit (Thermo scientific, Lithuania, EU). The final dsRNAs were dissolved in nuclease-free water. The size of the dsRNA products was determined with 1% agarose gel electrophoresis and was quantified using a spectrophotometer.

In this study, leaf-discs containing dsRNA-*TcCPR* were used to knock down the expression of *T. cinnabarinus TcCPR*. Cowpea leaves were cut to a 1.5 cm diameter feeding arena and incubated at 60 °C in lab oven for 3 minutes for dehydration. Then, the leaves were treated with DEPC-water, dsRNA-*GFP*, or dsRNA-*TcCPR* (1000 ng/μl) for 5 hours. After fully absorbed, the leaves were put on wet filter paper. Thirty female adults (3–5d old and starved for 24 hours) were placed on each leaf-disc. The mites were reared under controlled growth conditions: 26 ± 1 °C, 35%–55% (RH), and 14:10 (L:D) photoperiod. After feeding for 48 hours, the mites were collected to determine the reduction of *TcCPR* transcription levels using qRT-PCR. The qRT-PCR primers (Supplementary Table S1) were designed according to a separate region of *TcCPR* used for RNAi, to avoid hampering the qRT-PCR reactions. To further reveal the relationship of transcription levels between *TcCPR* and P450s, we have also detected the expression levels of two P450 genes (*CYP389B1* and *CYP392A26*) at 48 h post-feeding of dsRNA-*TcCPR*, which were over-expressed in FeR strain^40^. The qRT-PCR primers of *CYP389B1* and *CYP392A26* were also shown in Supplementary Table S1.

### Determination of P450s activity after RNAi

Total protein contents of the enzyme solutions were determined by the Bradford method using bovine serum albumin as the standard^55^. P450 activities were tested according to Shang’s method^56^. The 200 female adult mites at 48 h post-feeding of dsRNA-*TcCPR* were homogenized in 1.5 mL phosphatic buffer solution (PBS) (0.1 mol L^−1^, pH 7.8) on ice and were centrifuged at 10,000 g for 15 min at 4 °C. Then, the supernatants were extracted for testing. Using nitroanisole as the substrate, the detailed procedure was described by Wang *et al.*^57^. The specific activity was determined according to nitrophenol standard curve and protein concentration of enzyme source. The experiments were repeated for three times, and the average was obtained from the three repeated data.

### Bioassays with fenpropathrin after RNAi

In this study, fenpropathrin was dissolved in acetone. Sub-lethal doses of fenpropathrin (LC_30_ and LC_50_ of the SS and FeR strains, respectively)^32^ were applied for the bioassays in FeR strain. We also adopted the RCV method described above and the detailed bioassay procedure was described by Feng *et al.*^29^. Thirty treated female mites were transferred into the acaricide-coated centrifuge tubes; each dose was performed in three replicates, including acetone as control. The mites were checked under an anatomical microscope after 24 h rearing at 26 ± 1 °C; 35%–55% (RH); 14:10 (L:D) photoperiod. Mites showing immobility or with legs irregularly trembling were considered dead. Mean and standard errors were obtained from at least three independent bioassays.

### Statistical analysis

Data analyses were carried out using SPSS 19.0 (SPSS Inc., Chicago, USA). The relative quantities in SS and FeR strains were determined by independent-sample *t*-test with a *p*-value < 0.05. The differences on expression levels of CPR among four developmental stages, expression levels of CPR after induction, RNAi knockdown efficiencies, mortality rates, and specific activities of P450s were analyzed by one-way analysis of variance (ANOVA), followed by Duncan’s multiple tests, *p*-value < 0.05.

## Additional Information

**How to cite this article**: Shi, L. *et al.* Silencing NADPH-cytochrome P450 reductase results in reduced acaricide resistance in *Tetranychus cinnabarinus* (Boisduval). *Sci. Rep.* **5**, 15581; doi: 10.1038/srep15581 (2015).

## Supplementary Material

Supplementary tables

## Figures and Tables

**Table 1:** The specific activities of P450s after dsRNA-*TcCPR* feeding.

Treatments	Specific activity (nmol/mg pro.min^−1^)^1^
DEPC-water	8.7060 ± 0.6556^a^
dsRNA-*GFP*	8.6308 ± 0.84449^a^
dsRNA-*TcCPR*	2.1323 ± 1.0698^b^

^1^Values within a column followed by a different letter are significantly different according to Duncan’s multiple tests (*P* < 0.05). i.e. No statistical difference between “a” and “a”; significant difference between “a” and “b”.
