# Supplementary material for: Silencing NADPH-cytochrome P450 reductase results in reduced acaricide resistance in Tetranychus cinnabarinus (Boisduval)
Source: Sci Rep. 2015 Oct 23;5:15581. doi: 10.1038/srep15581 (PMC4616063; doi:10.1038/srep15581)
Supplement: Supplementary tables [file srep15581-s1.pdf]

Supplementary Information

Prepared for Publication in **Scientific Reports**

Address Correspondence to:

Dr. Lin He
Key Laboratory of Entomology and Pest Control Engineering
College of Plant Protection
Southwest University
Chongqing, China
Phone: +86-23-68251541
Fax: +86-23-68251514
E-mail :helinok@vip.tom.com

Dr. Qiang Xu
Department of Biology
ACU Box 27868
College of Arts and Sciences
Abilene Christian University
Abilene, Texas 79699 U.S.A.
Phone: +1-325-674-4883
Fax: +1-325-674-2009
E-mail : qxx07a@acu.edu

Silencing NADPH-cytochrome P450 reductase results in reduced acaricide resistance in *Tetranychus cinnabarinus* (Boisduval)

Li Shi[1], Jiao Zhang[1], Guangmao Shen[1], Zhifeng Xu[1],Peng Wei[1], Yichao Zhang[1],Qiang Xu[*2]and Lin He[*1]

[1]Key Laboratory of Entomology and Pest Control Engineering, College of Plant Protection, Southwest University, Chongqing, China;

[2]Department of Biology, Abilene Christian University, Abilene, Texas, U.S.A.

[*] To whom correspondence should be addressed

Table S1. Primers used for gene cloning, RNAi and qRT-PCR analysis

Genes	GenBank No.	Primer Function	Primer sequences (5' to 3')
GFP	ACY56286	RNAi	F: taatacgactcactatagggGCAGTTCTTGTTGAATTAGATG<br>R: taatacgactcactatagggTTTGGTTTGTCTCCCATGATG
TcCPR	KP710970	RNAi	F: taatacgactcactatagggTGAAGCTGGAGATCATCTTG<br>R: taatacgactcactatagggATCCATTCAGAGTACAAGGC
TcCPR	KP710970	Cloning full length	F: ATGGAAGAATCGCCTAATCAAGGAT<br>R: CTAACTCCACACATCAGCAGAATAT
TcCPR	KP710970	qRT-PCR	F: GGGTGATGATGATGCAAACA<br>R: ACGATAGGTGCCAAGAATGG
CYP389B1	KF770839	qRT-PCR	F: ACGAGCCATGAATCCGTTAC<br>R: TGATCGGTGTGCTTCTCAAG
CYP392A26	KF770838	qRT-PCR	F: CGTGAACACAGACGGTTGTC<br>R: GCGACCAAATAAAAGAGCTGA
RPS18	FJ608659	qRT-PCR	F: ACGTGCTGGTGAACTTACCGAAGA<br>R: TGCCTATTCAAGAACCAAAGTGGG
α-TUB	FJ526336	qRT-PCR	F:ACTACGCTCGTGGCCACTATACAA<br>R:ACCAGATCCAGTTCCACCTCCAAA

Table S2. Sequences used for phylogenetic analysis

Genes	Gene ID or GenBank No.	Species
AaCPR	XP_001656715	*Aedes aegypti*
OsCPR	ACL01092	*Ochlerotatus sollicitans*
AfCPR	ABO77954	*Anopheles funestus*
DmCPR	AGB92664.1	*Drosophila melanogaster*
MdCPR	AAA29295	*Musca domestica*
BmCPR	NP_001104834	*Bombyx mori*
RhCPR	XP_002423980	*Pediculus humanus corporis*
CfCPR	EFN67037	*Camponotus floridanus*
NlCPR	KF591574	*Nilaparvata lugens*
ClCPR	AFD50507	*Cimex lectularius*
HaCPR	ADK25060	*Helicoverpa armigera*
AgCPR	AAO24765	*Anopheles gambiae*
TuCPR	tetur18g03390	*Tetranychus urticae*
MoCPR	XP_003740943	*Metaseiulus occidentalis*
RpCPR	JAA59930	*Rhipicephalus pulchellus*
IsCPR	XP_002400171	*Ixodes scapularis*
BtCPR	AAI03400	*Bos Taurus*
RnCPR	NP_113764.1	*Rattus norvegicus*
HsCPR	NP_000932	*Homo sapiens*
